# BRAZILIAN ORTHOPEDISTS' OPINIONS AND PERCEPTIONS ON FEMOROACETABULAR IMPINGEMENT

**DOI:** 10.1590/1413-785220162406162400

**Published:** 2016

**Authors:** Leandro Ejnisman, Moin Khan, Olufemi Rolland Ayeni, Mohit Bhandari, Helder de Souza Miyahara, Jose Ricardo Negreiros Vicente

**Affiliations:** 1. Universidade de São Paulo, Faculdade de Medicina (School of Medicine), Hospital das Clínicas, Instituto de Ortopedia e Traumatologia, São Paulo, SP, Brazil; 2. McMaster University, Department of Surgery, Division of Orthopaedics, Hamilton, Ontario, Canada.

**Keywords:** Surveys and questionnaires, Physical examination, Hip, Femoroacetabular impingement.

## Abstract

**Objective:**

To assess the opinion of Brazilian orthopedists surgeons on the diagnosis and treatment of femoroacetabular impingement (FAI).

**Methods:**

A questionnaire was sent to several orthopedic societies around the world, including the *Sociedade Brasileira de Ortopedia e Traumatologia* (SBOT). This questionnaire was sent electronically and included questions on many topics related to FAI.

**Results:**

253 Brazilian orthopedists responded the questionnaire. Sixty-eight point nine percent worked in private practice and 23.1% in academic institutions. Pain during hip rotation was the most important finding in the clinical history according to 81.8% of the respondents and the anterior impingement sign was the most important finding in the physical examination according to 88.2%. Initial treatment was physiotherapy according to 86.2%. Surgical treatment was hip arthroscopy according to 38.8%, and via surgical hip dislocation for 14.7%.

**Conclusion:**

Brazilian orthopedists' opinions on FAI are similar to their international colleagues. There is considerable discrepancy in the answers provided, demonstrating a need for future investigation on FAI, in order to institute proper treatment and diagnosis protocols. Level of Evidence V. Expert Opinion.

## INTRODUCTION

Femoroacetabular impingement (FAI) is recognized as a cause of hip pain and as a predisposing factor for early-onset osteoarthritis in young patients.^1^ ^,^ ^2^ This disease is characterized by abnormal conflict between the femoral head and the acetabulum due to abnormal anatomy of the femoral head-neck junction (cam type), which is aspherical, and/or acetabular overcoverage (pincer type), resulting in injury to the labrum and articular cartilage.^3^ ^,^ ^4^

Treatment is initially conservative, and in cases of failure surgery is indicated.^5^ Surgical correction can be performed via an open or arthroscopic approach. However, there are few studies with adequate documentation on clinical and radiographic indications for the surgical correction of impingement, both via arthroscopy and via open surgery. Moreover, there is insufficient data to determine the natural history of femoroacetabular impingement.^6^

Given the shortage of scientific data on FAI incidence, epidemiology, prevalence and treatment methods, it is essential to understand the perceptions of orthopedic surgeons to serve as a guideline for future research and to better understand the treatment indications. This study aimed at comparing the opinion of Brazilian orthopedists with orthopedists around the world on the diagnosis, treatment and scientific evidence of FAI by means of a questionnaire.

## MATERIAL AND METHODS

This study was approved by the local scientific committee (identification 13-404). A group formed by a statistician and orthopedic surgeons was responsible for determining key areas of interest to be reached. This group was called IN-FOCUS (InterNational Femoroacetabuar Impingement Optimal Care Update Survey).^7^ Prior orthopedic questionnaires were reviewed to ensure that all the items were appropriate and understood.^8^ The questions were adapted to examine the respondent's level of understanding in relation to diagnosis, surgical indication (arthroscopic or open), and the scientific data from the literature on femoroacetabular impingement. The survey involved a 'redundancy sample' in which several new surgeons were interviewed until no new item was required in the questionnaire.^9^

The survey was pretested to guarantee its validity with an independent group of four orthopedic surgeons specialized in treating hip disorders in young adults and to ensure that the questionnaire was tenable in the search for perceptions related to FAI.

Sections related to epidemiology, treatment options, diagnosis and quality of available scientific evidence were refined through the surgeons' feedback, seeking an improvement in content, ease of comprehension and understanding of the survey.

Invitations were sent by email to the members of several international orthopedic societies, including the *Sociedade Brasileira de Ortopedia e Traumatologia* [Brazilian Society of Orthopedics and Traumatology (SBOT)]. The survey was conducted through the SurveyMonkey website. The invitations were resent twice two weeks apart in order to increase the response rate. Restrictions were applied to ensure that each partner would answer the questionnaire only once.

### Statistical analysis

All the answers were organized and analyzed on the actual SurveyMonkey website. Categorical data were presented as percentages. Since some questions had multiple correct answers, not all questions add up to 100%. In addition, the respondents could skip questions without answering them.

## RESULTS

### Demographic results

Two hundred fifty-three members of the SBOT answered the questionnaire, representing 28.1% of international responses. In the international study, most of the orthopedists who answered the questionnaire were from Europe (40.7%), followed by South America (29.3%) and North America (14.0%). Most of the Brazilian respondents work in private practice (68.9%) and in academic institutions (23.1%). Table 1 shows the answers of the orthopedists from around the world compared to the Brazilian answers.

**Table 1:**
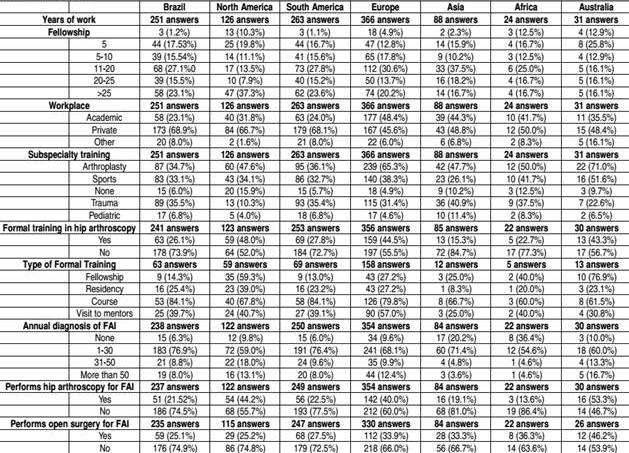
Demographics.

### Clinical evaluation

The most important finding in the clinical history of a patient with FAI is pain during hip rotation according to 81.8% of respondents, followed by groin pain (50.7%). The most important finding in the physical examination is the anterior impingement sign (pain upon internal rotation-adduction-flexion) according to 88.2% of physicians, followed by the C sign (33.5%).

In the respondents' opinion radiographic confirmation of the diagnosis is achieved through magnetic resonance imaging (75.9%), radiography alone (35.0%), computed tomography (26.6%) and intra-articular injection of anesthetic (9.85%). In the diagnosis of cam type FAI, the most important radiographic measurement was considered: the head-neck offset (40.9%) and the alpha angle (37.4%). In the diagnosis of pincer type FAI, the most important radiographic measurement was considered: crossover sign (43.84%), center-edge angle (30.0%) and acetabular inclination (26.6%); 21.2% of respondents were uncertain about the answer. Table 2 shows the perceptions of Brazilian and international orthopedists on the diagnosis of FAI.

**Table 2:**
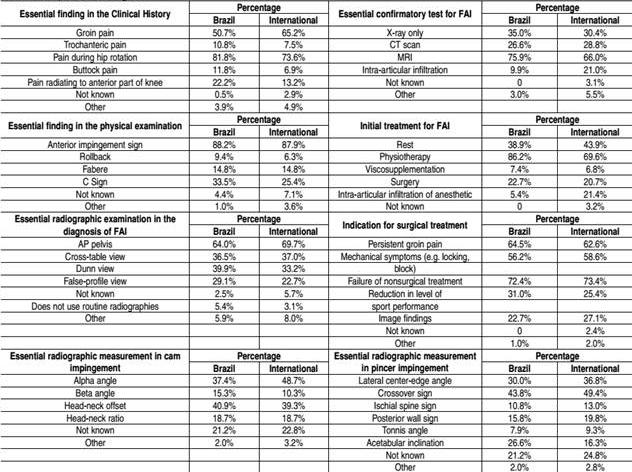
Perceptions on the diagnosis.

Most of the Brazilian surgeons diagnosed 1-30 cases of FAI in one year (76.9%), only 7.98% diagnosed over 50 cases and 6.30% did not diagnose any case. Fifty-one (21.5%) Brazilian surgeons perform hip arthroscopy, and 59 (25.1%) perform open surgery for correction of FAI. Surgeons who perform hip arthroscopy recorded the following volumes: fewer than 10 arthroscopies per year (41.1%), between 10 and 100 cases per year (54.9%) and more than 100 cases (3.9%). Among the surgeons who perform open correction, 68.3% operated fewer than 10 cases in a year and 5% operated more than 100 cases. Orthopedists who perform more than 50 hip arthroscopies per year (6 surgeons) have been in clinical practice between 11 and 20 years (50%), and between 20 and 25 years (33.3%). One hundred percent of the surgeons in this high volume group work in private practice.

After the diagnosis of FAI, 86.2% of orthopedists start treatment with physical therapy. Rest is prescribed by 38.9%, surgery by 22.6%, and viscosupplementation by 7.4%. The main surgical indication reported was failure of conservative treatment (72.4%), persistent hip pain (64.5%), mechanical symptoms in the hip (56.2%), and reduced level of sporting activity (31.0%). Surgeons performing more than 50 arthroscopies per year indicated surgery at an earlier stage. Of these surgeons with a higher volume, 66.7% considered the initial treatment of FAI to be surgical.

Brazilian surgeons treat FAI as follows: arthroscopy (38.8%), surgical dislocation (14.7%), combined arthroscopic and open techniques (9.4%), both arthroscopic and open technique (22.9%). Table 3 shows the Brazilian surgeons' perceptions on FAI treatment and surgical technique.

**Table 3:**
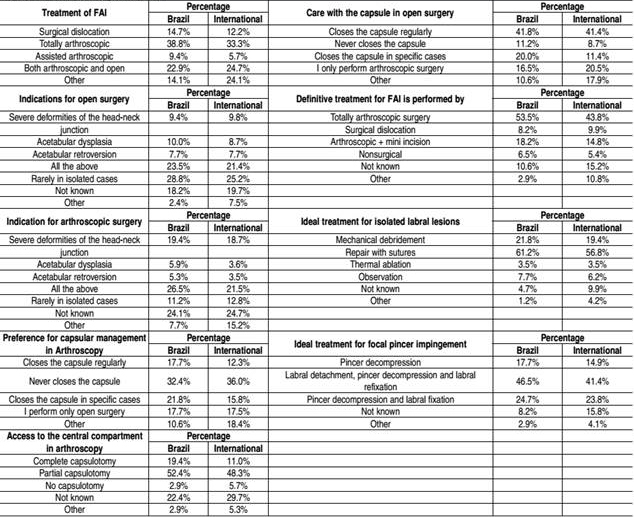
Perceptions on surgical treatment.

### Clinical outcome assessment

Clinical scores are regarded as the best method for assessing the outcome of FAI surgery by 66.9% of respondents. Other reported methods were: cartilage analysis by magnetic resonance (39.6%), radiographic correction of FAI (31.8%), gait analysis (13.6%) and cartilage degradation markers (12.3%). Radiographic parameters used to assess FAI correction were: degenerative alterations (44.2%), head-neck offset (40.9%), alpha angle (39.6%), crossover sign (29.2%) and center-edge angle (22.7%). The clinical parameters used were pain relief (76.0%), impingement sign (53.9%), range of motion (51.3%) and return to sports (48.0%). Some of the surgeons do not use clinical scores (37.0%). The scores used varied considerably: Harris Hip Score (23.4%), WOMAC (18.8%), Modified Harris Hip Score (15.6%), iHOT-33 (12.3%), and others.

### Scientific evidence

The respondents disagreed on the scientific evidence regarding the best clinical test for diagnosing FAI: 24.4% considered the evidence strong, 29.4% considered the evidence moderate and 25.0% considered the evidence weak. Similar data were reported in relation to radiographic diagnosis: 31.9% considered the evidence strong, 31.9% considered the evidence moderate and 13.7% considered the evidence weak. Evidence for the use of intra-articular diagnostic injections was considered even less appropriate: strong (20.0%), moderate (25.0%), weak (19.4%) and very weak (11.9%).

The surgeons also considered the evidence of conservative treatment inadequate: strong (15.0%), moderate (26.9%), weak (25.6%), very weak (16.2%). Most of the respondents (53.8%) believed that there is evidence to consider one FAI correction technique superior to the other. Evidence of osteoplasty in the treatment of cam impingement was considered strong by 32.9%, moderate by 29.7% and weak by 12.0% of the orthopedists. On the other hand, evidence of correction of pincer impingement was considered strong by 25.9%, moderate by 36.1% and weak by 17.0%. Evidence of the treatment of labral lesions was considered strong by 33.5%, moderate by 26.6% and weak by 17.7% of respondents.

Evidence of positive results following FAI surgery was considered very strong by 5.84%, strong by 31.2%, moderate by 35.0% and weak by 12.3% of surgeons. Evidence of the relationship between FAI and the development of hip osteoarthritis was considered very strong by 22.1%, strong by 37.0%, moderate by 24.0% and weak by 6.5% of orthopedists. Seventy-nine percent agreed that hip osteoarthritis is a negative predictor of post hip arthroscopy results.

## DISCUSSION

In the present study we evaluated Brazilian orthopedists' opinions of FAI, and compared them with colleagues around the world. The Brazilian answers were very similar to international answers, demonstrating that Brazil is at the forefront of hip preservation surgery.

In analyzing the academic background of the questionnaire respondents we can see that hip pain treatment is performed mainly by orthopedists specialized in arthroplasty, sports medicine and trauma. We also found that only 26.1% have formal training in hip arthroscopy. These figures are similar to the rest of South America, Asia and Africa, but differ from the US, Europe and Australia, where almost half of the respondents have formal training in hip arthroscopy. In Brazil, much of this training involves courses and visits to mentors; residency and fellowship training is much less common. A possible explanation for this lack of formal training is the shortage of healthcare facilities with hip arthroscopies available in the public system. Most arthroscopies in Brazil are performed in private practice, where there are fewer residents.

The Brazilians' opinions on the diagnosis and initial treatment of FAI are very similar to the international responses. Groin pain is considered an essential finding in the patient's clinical history and the anterior impingement sign essential in the physical examination. As regards diagnostic imaging, 75.9% consider MRI essential. A difference found between Brazilians and foreigners is the use of intra-articular infiltration as a diagnostic method. Among Brazilian 9.9% use infiltration, while 21.0% of foreigners use this method. Most Brazilians and foreigners start the treatment with rest and physical therapy, while approximately 20% start with surgery. The main surgical indications are failure of nonsurgical treatment, persistent groin pain and mechanical symptoms. The conservative treatment of FAI remains highly controversial. There is no clear indication whether it should be performed, how and for how long.^10^ Future surveys should seek these answers.

The answers regarding surgical treatment reflect the uncertainties found in the literature. Indications for open or arthroscopic treatment are not yet clear. Recent studies indicate adequate corrections via the arthroscopic approach, but the open approach would be more effective in cases of more posterior cam deformity.^11^ Brazilian orthopedic surgeons tend to opt for arthroscopic treatment of FAI (38.8% of orthopedists use arthroscopy while 14.7% use surgical dislocation). New questionnaires could assess the cause of this discrepancy. We believe that the decision on which technique to use is not only related to the surgeon's option, but also to the availability of materials at each facility.

The evaluation of the surgeons' opinion on scientific evidence related to FAI shows that many aspects related to the treatment of this condition still require further scientific research. In analyzing the international data of the IN-FOCUS study, we believe that knowledge of FAI is at a "turning point".^7^ According to McCulloch et al.,^12^ the evolution of surgical techniques follows a pre-established innovation cycle model. Under this concept, new surgical techniques are developed by pioneers, usually trendsetters who develop the basic concepts of the technique. Over time, indications expand, there is a refinement of the technique and clinical studies begin. At this moment, there is a rapid turnaround with an increase in the number of surgeons using the technique. We believe that FAI is at this "turning point".

Our study has some limitations. Questionnaires were sent in English, which may have limited the response of some Brazilian orthopedists not familiar with this language. Furthermore, some physicians may not be familiar with survey webpages such as SurveyMonkey, which may have curtailed some surgeons, particularly the older ones. In the future, similar Brazilian surveys should be conducted in Portuguese either face to face or by conventional mail with the aim of reducing these biases.

## CONCLUSION

The diagnosis and treatment of FAI has been growing exponentially in Brazil and in the world, but diagnostic criteria, surgical indications and treatment methods remain controversial. Perceptions of Brazilian orthopedists are similar to the opinions of international surgeons.
